# Assessment of Cyclophilin A levels in early and late pregnancy among women with preeclampsia

**DOI:** 10.3389/fphys.2026.1687130

**Published:** 2026-03-10

**Authors:** Neset Gumusburun, Selim Gulucu, Sebahattin Celik, Sercan Serin

**Affiliations:** 1 Department of Obstetrics and Gynecology, Istanbul Aydin University, Istanbul, Türkiye; 2 Department of Obstetrics and Gynecology, Gaziosmanpasa University, Tokat, Türkiye; 3 Department of Obstetrics and Gynecology, Balikesir State Hospital, Balikesir, Türkiye; 4 Department of Obstetrics and Gynecology, Samsun Training and Research Hospital, Samsun, Türkiye

**Keywords:** Cyclophilin A, first trimester, third trimester, preeclampsia, pregnancy

## Abstract

**Background:**

Pre-eclampsia is one of the leading causes of maternal-fetal morbidity. Cyclophilin A, which plays a role in inflammation and vascular dysfunction, may contribute to the pathogenesis of pre-eclampsia; however, trimester-specific levels have not been adequately investigated. The aim of this study was to evaluate serum Cyclophilin A levels in women with pre-eclampsia in the first and third trimesters and to examine its association with preeclampsia across different trimesters.

**Methods:**

This prospective case–control study was conducted with 120 pregnant women without prior medical conditions. Serum samples were collected during the first trimester in all participants and during the third trimester after diagnosis in women who developed preeclampsia. Cyclophilin A levels were measured and compared between preeclampsia and control groups, as well as early- and late-onset preeclampsia subgroups based on gestational age at delivery.

**Results:**

No statistically significant differences were observed between the preeclampsia and control groups in terms of maternal age, gravidity, or parity (p > 0.05). However, serum cyclophilin A concentrations were markedly elevated in the preeclampsia group compared to healthy controls during both the first and third trimesters (p < 0.05). Subgroup analysis further revealed that patients with early-onset preeclampsia exhibited significantly higher CYPA levels in both trimesters when compared to those with late-onset preeclampsia (p < 0.05).

**Conclusion:**

Cyclophilin A levels were higher in the first trimester in women who later developed preeclampsia and remained elevated in the third trimester in women with established disease. These findings suggest a trimester-specific association between cyclophilin A and preeclampsia, warranting further investigation.

## Introduction

Preeclampsia (PE) is a pregnancy-specific hypertensive condition that presents after the 20th week of gestation and is characterized by new-onset hypertension after 20 weeks of gestation, with or without proteinuria, in previously normotensive individuals. According to current ACOG diagnostic criteria, preeclampsia may also be diagnosed in the absence of proteinuria when new-onset hypertension is accompanied by signs of maternal end-organ dysfunction (Erez et al., 2022). From a clinical point of view, hypertension is defined as a condition in which systolic blood pressure (SBP) reaches 140 mmHg or above or diastolic blood pressure (DBP) is measured at 90 mmHg or above (Obstetricians, 2013). PE is a pregnancy-specific condition associated with increased maternal and perinatal morbidity and mortality, affecting approximately 5%–7% of all pregnancies (Mol et al., 2016). Although the underlying mechanisms are not fully elucidated, current evidence suggests that abnormal trophoblastic invasion and inadequate placental perfusion contribute to its pathogenesis. Impaired placental development, elevated oxidative stress, and increased apoptosis and necrosis within the placenta lead to endothelial dysfunction, which subsequently manifests as maternal hypertension and proteinuria (Gülücü et al., 2021). Although preeclampsia is clinically diagnosed after 20 weeks of gestation, its pathophysiological processes begin much earlier, particularly during placentation in the first trimester. Therefore, biomarkers measured in early pregnancy may reflect underlying placental and endothelial alterations that precede the clinical manifestation of the disease. These processes are closely linked to the release of stress-responsive proteins and inflammatory mediators into the maternal circulation.

Cyclophilin A (CYPA) is a member of the immunophilin protein family and acts as a high-affinity binding site for the immunosuppressive drug cyclosporine (Celik et al., 2020). It plays a crucial role in various biological processes, including intracellular signaling, regulation of inflammation, and apoptosis. Elevated levels of CYPA have been documented in inflammatory and oxidative stress-related conditions, such as coronary artery disease and chronic obstructive pulmonary disease (Zhang et al., 2018; Ohtsuki et al., 2017). CYPA is also known to participate in vascular remodeling by enhancing inflammation and promoting the proliferation of vascular smooth muscle cells (Satoh et al., 2010). Recent advances in obstetric research highlight the clinical value of identifying early biomarkers reflecting underlying pathophysiological changes before the clinical onset of PE (Rana et al., 2019; Redman and Sargent, 2009). Traditional diagnostic methods usually detect the disease after maternal or fetal risk has occurred (Magee et al., 2014). Therefore, there is increasing interest in circulating biomarkers that may allow earlier risk stratification (Sibai, 2013). Cyclophilin A, released in response to oxidative stress and endothelial damage, is an example (Jin et al., 2000). In addition, Hu et al. demonstrated that CYPA inhibits trophoblast migration and invasion, suggesting a possible contribution to the pathophysiology of preeclampsia (Hu et al., 2020).

Although increased cyclophilin A levels in preeclampsia have been reported in previous studies, most available data are derived from cross-sectional or single time-point measurements. The present study was designed to evaluate trimester-specific differences in cyclophilin A levels by comparing early pregnancy measurements with levels obtained after the clinical manifestation of the disease in late pregnancy within a prospectively followed cohort. By comparing early- and late-onset preeclampsia, this study aims to provide further insight into the temporal relationship between cyclophilin A levels and the development of preeclampsia.

## Materials and methods

This study was designed as a prospective cohort study with a nested case–control comparison and included pregnant women who applied to the Samsun Training and Research Hospital Gynecology and Obstetrics outpatient clinic between October 2021 and October 2022. Before the study, approval was obtained from the local ethics committee on 23.09.2021 with project number KAEK 2021/419. The study was conducted in accordance with the principles of the Declaration of Helsinki, and informed consent was obtained from all participants. Pregnant women were prospectively followed throughout gestation, and participants were consecutively recruited during routine antenatal visits. Women who subsequently developed preeclampsia constituted the case group, while normotensive pregnant women with uncomplicated pregnancies, recruited during the same period, served as the control group. The consecutive inclusion of participants was intended to minimize selection bias. Clinical follow-up was prospective for all participants; however, biological sampling was not designed as a fully balanced longitudinal protocol across all individuals. Therefore, trimester-specific comparisons were performed at the group level; within-subject paired analyses were not applicable to the full dataset.

### Patient recruitment

During the study period, pregnant women undergoing routine first-trimester antenatal evaluation were prospectively followed until delivery. Women who subsequently developed preeclampsia constituted the preeclampsia group, while the control group consisted of normotensive women with singleton pregnancies who experienced no obstetric complications throughout gestation and delivered at term. Serum samples obtained at predefined gestational periods were included in the final analysis; however, the sampling scheme was not designed as a fully balanced longitudinal protocol across all participants. The first trimester was defined as 11–13+6 weeks of gestation. Third-trimester serum samples were obtained from both the preeclampsia and control groups during late pregnancy as part of routine antenatal follow-up, and all samples were collected prior to delivery. As sampling was performed within routine clinical care, exact gestational weeks for third-trimester samples were not uniformly recorded and are therefore reported at the trimester level. Exact gestational weeks and the interval from diagnosis to sampling could not be uniformly retrieved from hospital records; therefore, precise timing relative to diagnosis or delivery could not be reported.

Maternal serum samples were obtained during routine antenatal visits in the first trimester from all participants and stored at −80 °C until biochemical analysis. All participants were prospectively monitored throughout pregnancy, and obstetric outcomes were systematically recorded.

Inclusion criteria for the preeclampsia group were singleton pregnancy and a new diagnosis of preeclampsia according to established diagnostic criteria. The control group consisted of women with singleton pregnancies who remained normotensive throughout gestation and did not develop any obstetric or medical complications. Apart from the presence of preeclampsia, the same inclusion and exclusion criteria were applied to both groups. Exclusion criteria for both groups included multiple pregnancies, fetal anomalies, chronic hypertension, vascular disease, autoimmune disorders, renal disease, and diabetes mellitus. Diabetes mellitus was defined as both pregestational diabetes and gestational diabetes mellitus diagnosed during pregnancy. Demographic data (age, gravidity (number of pregnancies), parity, gestational age (weeks), body massindex and mode of delivery), blood parameters (haemoglobin, platelet, ALT, AST and CYPA) and neonatal information (birth weight, Apgar score at 1 and 5 min, presence of meconium in amniotic fluid and need for neonatal intensive care unit) were evaluated. Participants with HELLP syndrome, thrombocytopenia (<100,000/µL), or significantly elevated liver enzymes (AST/ALT ≥2× upper limit of normal) were excluded from the study.

Gestational age was established using the first day of the last menstrual period (LMP), or, in cases where LMP data were not available, based on first-trimester ultrasound measurements. The diagnosis of preeclampsia is made in a previously normotensive pregnant woman, after the 20th week of pregnancy, with systolic blood pressure ≥140 mmHg or diastolic pressure ≥90 mmHg in two separate measurements taken at least 4 hours apart, and proteinuria defined as ≥+1 on a dipstick test or ≥300 mg in a 24-h urine sample (Obstetricians ACo, 2020) or in the absence of proteinuria, according to current ACOG diagnostic criteria, when new-onset hypertension is observed with maternal end-organ dysfunction (Erez et al., 2022). The preeclampsia group was stratified into early-onset (before 34 weeks of gestation) and late-onset (34 weeks or later) subgroups according to the gestational age at the time of delivery (Rode et al., 2021). This delivery-based definition was used because the exact timing of clinical diagnosis could not be uniformly retrieved from medical records. Early-onset and late-onset preeclampsia were defined based on gestational age at delivery rather than the timing of diagnosis. Preeclampsia cases were not further subclassified according to severity (with or without severe features), as the primary aim of the study was to evaluate cyclophilin A levels in relation to the presence and timing of preeclampsia rather than disease severity. Although serum samples were obtained at more than one gestational time point from some participants, the analyses were not designed to evaluate within-subject longitudinal changes over time.

### Biochemical analysis

For biochemical analysis, the collected blood specimens were centrifuged at 4,000 revolutions per minute for 8 min to isolate the serum component. Approximately 4 mL of venous serum was stored at −80 °C. Following thawing, serum CYPA concentrations were determined using a commercially available enzyme-linked immunosorbent assay (ELISA) kit (Sun-Red Bio Company, Cat No. 201-12-0675, Shanghai, China). Results were expressed in nanograms per milliliter (ng/mL). All serum samples were analyzed in duplicate according to the manufacturer’s instructions. Laboratory personnel performing the ELISA assays were blinded to the clinical status of the participants. The analytical sensitivity of the assay was reported by the manufacturer as <0.1 ng/mL. The intra-assay and inter-assay coefficients of variation were both reported to be <10%.

Serum cyclophilin A concentrations were measured using a commercially available ELISA kit according to the manufacturer’s instructions. The analytical performance characteristics of the assay, including detection range, analytical sensitivity, and intra- and inter-assay variability, were provided by the manufacturer. All samples were analyzed in duplicate, and mean values were used for statistical analysis. Laboratory personnel performing the assays were blinded to the clinical data. The ELISA kit was validated by the manufacturer for use in human serum samples. No samples were excluded as outliers, and all measured values were included in the analyses to reflect the biological variability of cyclophilin A levels.

### Statistical analysis

All statistical analyses were conducted using IBM SPSS Statistics software version 25. A p-value of less than 0.05 was accepted as the threshold for statistical significance. As continuous variables did not follow a normal distribution, normality was assessed and descriptive statistics were reported as median (minimum–maximum), with non-parametric statistical tests applied for group comparisons. As subgroup analyses involved small sample sizes, non-parametric statistical methods were used, and findings were interpreted as exploratory. Categorical variables were summarized using frequencies and percentages. As continuous variables did not follow a normal distribution, non-parametric statistical methods were used for all group comparisons. The Mann–Whitney U test was applied for continuous variables, and Pearson’s chi-square or Fisher’s exact test was used for categorical variables, as appropriate. No multivariable regression analysis was performed to adjust for potential confounding factors such as maternal age, body mass index, or gestational age. Although serum samples were obtained from the same participants at different stages of pregnancy, repeated-measures or longitudinal statistical models were not applied; instead, trimester-specific comparisons were performed as independent analyses in line with the exploratory design of the study. No formal adjustment for multiple comparisons was applied, as subgroup analyses were considered exploratory in nature. Given the wide distribution of cyclophilin A concentrations, non-parametric statistical methods were used for group comparisons. Maternal age did not meet the assumption of normal distribution and was therefore reported as median (minimum–maximum). No multivariable regression modeling was performed, as the study was not designed to develop an adjusted predictive model.

## Results

During the study period, 2,536 pregnant women underwent first-trimester evaluations. Among these, 61 women were diagnosed with preeclampsia and constituted the study group, while 59 women with uncomplicated pregnancies served as the control group. Therefore, serum samples collected during both the first and third trimesters from a total of 120 participants were included in the final analysis.

There were no statistically significant differences between the PE and control groups with respect to maternal age, gravidity, or parity. Median maternal age was 30 (range: 20–42) years in the preeclampsia group and 26 (range: 19–40) years in the control group (p = 0.623). Pre-pregnancy body mass index was significantly higher in women with preeclampsia compared to controls (median: 29 [20–48) vs. 26 [18–38], p = 0.002). In terms of neonatal outcomes, meconium-stained amniotic fluid was more frequently observed in the PE group, and the incidence of NICU admissions was also greater, although this latter difference did not reach statistical significance. Furthermore, both APGAR scores at 1 and 5 min, as well as birth weights, were significantly lower in the PE group. Neonatal birth weight was significantly lower in the preeclampsia group compared to controls (median: 2,770 g [870–3,890] vs. 3,560 g [2,500–4,500], p < 0.001). The 1-min Apgar score was lower in the preeclampsia group (median: 8 [4–9] vs. 9 [6–9], p < 0.001], as was the 5-min Apgar score (median: 9 [5–10] vs. 10 [8–10], p < 0.001). First-trimester serum cyclophilin A levels were significantly higher in women with preeclampsia compared to controls (median: 24.69 ng/mL [7.77–58.91] vs. 8.12 ng/mL [5.03–48.11], p < 0.001). Similarly, third-trimester cyclophilin A concentrations were elevated in the preeclampsia group (median: 21.12 ng/mL [8.15–110.23] vs. 7.35 ng/mL [6.06–18.12], p < 0.001). A detailed summary of the demographic characteristics and serum biomarker levels is presented in Table 1.

**TABLE 1:** Maternal demographics and biochemical profiles of study participants.

Variable	Preeclampsia (n = 61)	Control (n = 59)	p
Age	30 (20–42)	26 (19–40)	0,623
Gravidity	2 (1–5)	2 (1–5)	0,193
Parity	0 (0–4)	1 (0–4)	0,117
BMI	29 (20–48)	26 (18–38)	**0,002**
Type of birth			**<0,001**
C/S VD	42 (% 68,9) 19 (% 31,1)	20 (% 33,9) 39 (% 66,1)	
Presence of meconium	15 (% 24,6)	4 (% 6,8)	**0,015**
NICU	10 (% 16,4)	3 (% 5,1)	0,089
Birth weight	2,770 (870–3,890)	3,560 (2,500–4,500)	**<0,001**
Apgar 1. Min	8 (4–9)	9 (6–9)	**<0,001**
Apgar 5. Min	9 (5–10)	10 (8–10)	**<0,001**
First trimester cyclophilin A (ng/mL)	24.69 (7.77–58.91)	8.12 (5.03–48.11)	**<0,001**
Third trimester cyclophilin A (ng/mL)	21.12 (8.15–110.23)	7.35 (6.06–18.12)	**<0,001**

* Continuous variables are presented as median (minimum–maximum). Categorical variables are presented as number (%). Comparisons between groups were performed using the Mann–Whitney U test for continuous variables. Categorical variables were compared using the Pearson chi-square test or Fisher’s exact test, as appropriate.

** Abbreviations: VD: vaginal delivery, C/S: cesarean section, BMI: body mass index, NICU: neonatal intensive care unit.

***Bold values indicate statistically significant differences between groups (p < 0.05).

Within the preeclampsia cohort, subgroup analysis was performed on 61 patients. Of these, 28 delivered before 34 weeks of gestation and were categorized as early-onset PE, while 33 delivered at or beyond 34 weeks and were classified as late-onset PE. No statistically significant differences were observed between the two subgroups in terms of maternal age, gravidity, parity, body mass index, or smoking status. However, evaluation of neonatal outcomes revealed that the early-onset group had a significantly higher incidence of meconium-stained amniotic fluid and increased NICU admission rates. Additionally, birth weight and Apgarscores at both 1 and 5 min were significantly lower in this group. Among women with preeclampsia, first-trimester cyclophilin A levels were significantly higher in early-onset cases compared to late-onset cases (median: 46.68 ng/mL [8.12–58.91] vs. 16.26 ng/mL [7.77–53.91], p = 0.003). Third-trimester cyclophilin A levels showed a similar pattern, with higher concentrations in early-onset preeclampsia (median: 45.89 ng/mL [9.52–110.23] vs. 18.32 ng/mL [8.15–38.09}, p < 0.001). A summary of maternal demographics and biochemical parameters for these subgroups is provided in Table 2.

**TABLE 2:** Demographic and analytical findings among women with preeclampsia.

Variable	<34 weeks (early onset PE) (n = 28)	34 weeks and above (late-onset PE) (n = 33)	p
Age	30 (20–38)	27 (20–42)	0,077
Gravidity	2 (1–5)	1,5 (1–5)	0,193
Parity	1 (0–4)	0 (0–3)	0,342
BMI	30 (20–42)	28 (21–48)	0,296
Type of birth			**<0,001**
C/S VD	12 (% 42,9) 16 (% 57,1)	30 (% 90,9) 3 (% 9,1)	
Presence of meconium	13 (% 39,4)	2 (% 7,1)	**0,009**
NICU	10 (% 30,3)	0 (% 0)	**0,001**
Birth weight	2134,1 ± 705,86	3,060 ± 312,19	**<0,001**
Apgar 1. Min	8 (4–9)	8 (7–9)	0,091
Apgar 5. Min	9 (5–10)	9 (8–10)	0,120
First trimester cyclophilin A (ng/mL)	46.68 (8.12–58.91)	16.26 (7.77–53.91)	**0,003**
Third trimester cyclophilin A (ng/mL)	45.89 (9.52–110.23)	18.32 (8.15–38.09)	**<0,001**

* Continuous variables are presented as median (minimum–maximum). Categorical variables are presented as number (%). Comparisons between early- and late-onset preeclampsia groups were performed using the Mann–Whitney U test for continuous variables and the Pearson chi-square test or Fisher’s exact test for categorical variables, as appropriate. Subgroup analyses were considered exploratory.

**Abbreviations: VD: vaginal delivery, C/S: cesarean section, BMI: body mass index, NICU: neonatal intensive care unit.

***Bold values indicate statistically significant differences between groups (p < 0.05).

All subgroup analyses were exploratory and should be interpreted accordingly.

## Discussions

This study investigated the association between serum cyclophilin A (CYPA) levels and the development of preeclampsia by comparing measurements obtained during the first and third trimesters in women with and without the condition. To our knowledge, few studies have evaluated trimester-specific CYPA concentrations within prospectively followed populations. The results demonstrated that CYPA levels were significantly elevated in individuals diagnosed with PE compared to normotensive controls at both time points. Additionally, the subgroup analysis demonstrated that individuals diagnosed with early-onset preeclampsia (prior to 34 weeks of gestation) had markedly elevated CYPA levels in both the first and third trimesters when compared to those with late-onset PE (≥34 weeks of gestation). It should be emphasized that the present study was not designed as a predictive modeling study but rather as an exploratory analysis comparing cyclophilin A concentrations between groups. In this context, first-trimester cyclophilin A measurements were intended to capture early biological alterations that precede the clinical diagnosis of preeclampsia, rather than to reflect overt disease or to serve as a definitive predictive marker. Accordingly, the findings should be interpreted as hypothesis-generating and indicative of a potential temporal association, rather than as evidence of direct clinical predictive utility. Given that exact gestational weeks at third-trimester sampling could not be retrieved for all participants, the interpretation of third-trimester CYPA differences should be considered exploratory rather than definitive.

Cyclophilin A (CYPA) is an intracellular protein secreted by smooth muscle cells, macrophages, and platelets in reaction to increased oxidative stress. Its expression increases in the presence of vascular injury, promoting endothelial cell apoptosis and ultimately contributing to endothelial dysfunction (Tian-Tian et al., 2013). The involvement of endothelial dysfunction and inflammation in the pathogenesis of preeclampsia has been well established in prior research (Seizer et al., 2016; Wibowo et al., 2017). In pregnancies complicated by PE, inadequate trophoblastic invasion and failure to properly remodel the maternal spiral arteries lead to the formation of atherotic lesions and thrombi within the vascular lumen, resulting in placental hypoxia and ischemia (Vitoratos et al., 2012). Although cyclophilin A is not a placenta-specific protein, placental hypoxia and oxidative stress—hallmark features of preeclampsia—can stimulate its release from trophoblasts and vascular endothelial cells. Once released into the maternal circulation, cyclophilin A may reflect the maternal vascular response to placenta-driven oxidative stress and endothelial injury rather than representing a nonspecific systemic inflammatory marker.

İn our study, It is noteworthy that body mass index was significantly higher in the preeclampsia group. Obesity-related meta-inflammation and metabolic dysfunction are known contributors to endothelial dysfunction and the pathogenesis of preeclampsia (Lourenço and Guedes-Martins, 2025). Although we did not perform a specific analysis to evaluate the direct association between CYPA levels and BMI or other metabolic parameters, all women with pregestational or gestational diabetes mellitus were excluded to minimize metabolic confounding. Moreover, despite the absence of a significant difference in BMI between early- and late-onset preeclampsia subgroups, CYPA levels remained significantly higher in early-onset cases, suggesting that CYPA elevation may not be solely attributable to increased BMI. Because subgroup classification was based on gestational age at delivery rather than the precise timing of disease onset, the distinction between early- and late-onset PE in this study may not fully reflect underlying biological onset and should therefore be interpreted cautiously.

Earlier studies have reported increased cyclophilin A concentrations during the first trimester in women who subsequently developed pregnancy-related complications, including gestational diabetes, gestational hypertension, and preeclampsia. These observations suggest that cyclophilin A may reflect early pathophysiological alterations associated with adverse pregnancy outcomes, rather than serving as a definitive predictive biomarker (Shaaya et al., 2022; Wang et al., 2018). In a study conducted by Celik et al., which involved 92 participants, CYPA concentrations measured during the early second trimester were significantly higher in the PE group compared to healthy controls (Celik et al., 2020). Although elevated cyclophilin A levels in preeclampsia have been previously reported, the present study adds to the existing literature by examining trimester-specific CYPA levels in a prospectively followed cohort and by comparing early- and late-onset preeclampsia. Rather than characterizing the full longitudinal trajectory of CYPA throughout pregnancy, the study was designed to contrast early pregnancy measurements with levels obtained after the clinical manifestation of the disease. Previous studies investigating cyclophilin A in preeclampsia have primarily focused on single time-point measurements, assessing CYPA either in early pregnancy or in later gestational periods. While these studies provided important initial insights into the association between CYPA and preeclampsia, they did not address trimester-specific differences within prospectively followed populations. By examining cyclophilin A levels in early and late pregnancy in a prospectively followed cohort, the present study extends these findings and provides additional temporal context, without aiming to characterize a fully longitudinal trajectory across all stages of pregnancy. These associations were not adjusted for BMI or other metabolic factors, and may therefore partially reflect underlying inflammatory or metabolic influences rather than disease-specific pathways.

In the current study, cesarean delivery was observed more frequently among patients with PE. Furthermore, neonates born to mothers in the PE group exhibited lower birth weights and reduced APGAR scores at both the first and fifth minutes, along with an increased need for NICU admission. These perinatal findings are consistent with those reported in the studies by Celik et al. and Sun et al., where similar trends in delivery mode and neonatal outcomes were observed (Celik et al., 2020; Sun et al., 2019).

Early-onset preeclampsia is typically linked to more pronounced placental abnormalities, severe maternal and fetal clinical manifestations, and poorer pregnancy outcomes (Lisonkova and Joseph, 2013). In the present study, CYPA concentrations were found to be significantly higher in patients with early-onset PE compared to those with late-onset PE during both the first and third trimesters. Moreover, consistent with previous literature, early-onset cases exhibited a significantly higher incidence of meconium-stained amniotic fluid and increased NICU admissions. These patients also had neonates with lower birth weights, further supporting the association between early-onset PE and adverse perinatal outcomes. Overall, CYPA levels were associated with preeclampsia and were higher in women who developed early-onset disease. The present study was not designed to prioritize one preeclampsia subtype over the other, but rather to comparatively evaluate cyclophilin A levels in early- and late-onset preeclampsia within a prospectively followed study population. Given that early-onset preeclampsia is often associated with a more severe clinical course, the observed increase in cyclophilin A levels may also reflect disease severity rather than a distinct biological specificity. This possibility should be considered when interpreting subgroup differences.

In addition to early detection, assessing the severity and potential prognosis of preeclampsia is of critical clinical importance. However, research focusing specifically on the severity and progression of the disease remains limited. Wibowo et al. conducted a study comparing 23 individuals with severe preeclampsia to 16 normotensive pregnant controls and reported that serum CYPA levels were significantly higher in the severe PE group. Following this, Sun et al. investigated the relationship between CYPA gene polymorphisms and their expression levels in both serum and placental tissue among patients with severe preeclampsia. Their findings indicated that the genetic variants rs3735481 and rs9638978 of CYPA were significantly associated with severe PE among Chinese women (Sun et al., 2019). In the present study, however, patients were not categorized based on disease severity, and thus, no conclusions could be drawn regarding the correlation between CYPA levels and the clinical severity of preeclampsia.

Beyond its diagnostic potential, Cyclophilin A (CYPA) has been explored as a potential therapeutic target in experimental models of vascular disease due to its active involvement in oxidative stress and vascular injury pathways. Recent experimental studies have shown that inhibition of extracellular CYPA with specific antagonists, such as cyclosporine derivatives or anti-CYPA antibodies, attenuates vascular inflammation and endothelial dysfunction in animal models of hypertension and atherosclerosis (Nigro et al., 2011; Benschop et al., 2020). Given the overlapping pathophysiological mechanisms between these conditions and preeclampsia, future translational research could explore whether similar interventions may alleviate PE severity or delay its onset. Furthermore, elevated CYPA has been correlated with adverse outcomes in cardiovascular disease, raising the possibility of its use as a prognostic marker for long-term cardiovascular risk in women with a history of PE (Canti et al., 2010). As preeclampsia is increasingly recognized as a marker of maternal cardiovascular vulnerability later in life, cyclophilin A may represent a biomarker of interest for understanding postpartum pathophysiological trajectories. However, to clarify its role in this broader context, longitudinal studies extending into the postpartum period and beyond are required. Such studies may help to further characterize the temporal behavior of cyclophilin A following preeclampsia, rather than to establish direct diagnostic, therapeutic, or preventive clinical applications.

Several limitations of the present study should be acknowledged. First, cyclophilin A (CYPA) measurements were not obtained during the second trimester, nor were CYPA concentrations assessed in cord blood samples from patients with preeclampsia. The absence of second-trimester sampling was primarily attributable to financial constraints, as the study was conducted without external funding and the cost of ELISA kits limited the feasibility of additional sampling time points. The first and third trimesters were therefore deliberately selected to contrast early pregnancy measurements with levels obtained after the clinical manifestation of preeclampsia. Future longitudinal studies incorporating second-trimester assessments may provide a more comprehensive characterization of CYPA dynamics throughout gestation. Second, no formal *a priori* sample size or power calculation was performed. Although statistically significant differences in serum CYPA levels were observed between groups, suggesting that the available sample size was sufficient to detect meaningful associations, this limitation should be considered when interpreting the results. In addition, the sample sizes of the early- and late-onset preeclampsia subgroups were relatively small, limiting the statistical power to detect differences between these subgroups and supporting an exploratory interpretation of these findings. Third, multivariable analyses adjusting for potential confounding factors such as maternal age, body mass index (BMI), gestational age, and smoking status were not performed. Consequently, residual confounding cannot be excluded, and the observed associations should be interpreted with caution. In particular, the relationship between CYPA levels and BMI or other markers of metabolic dysfunction was not specifically evaluated, and obesity-related inflammation may have contributed to the observed differences in CYPA concentrations. Fourth, no correction for multiple comparisons was applied in subgroup analyses, which may increase the risk of type I error and should be considered when interpreting these results. In addition, early- and late-onset preeclampsia were classified based on gestational age at delivery rather than at the time of clinical diagnosis, and therefore the potential influence of gestational age at sampling on CYPA levels cannot be completely excluded. Finally, markers of disease severity were not systematically analyzed, and thus the contribution of preeclampsia severity to the observed differences in CYPA levels between early- and late-onset cases cannot be fully determined. Taken together, the exploratory design, limited subgroup sample sizes, absence of multivariable adjustment, and uncertainties related to longitudinal sampling restrict the interpretation of the findings. Accordingly, the results should be regarded as associative and hypothesis-generating rather than predictive or directly translatable into clinical practice. Furthermore, since early- and late-onset PE were classified according to gestational age at delivery, subgroup comparisons may be influenced by differences in delivery timing rather than true biological onset. Another limitation of the present study is the absence of multivariable adjustment for potential confounders such as BMI, maternal age, parity, or smoking status. Therefore, residual confounding cannot be excluded, and the findings should be interpreted as associative rather than predictive.

In conclusion, serum cyclophilin A levels were higher in the first trimester among women who subsequently developed preeclampsia and remained elevated in the third trimester in women with established disease. These findings indicate an association between cyclophilin A levels and preeclampsia across different stages of pregnancy. However, given the exploratory design of the study and the absence of predictive or longitudinal modeling, the results should be interpreted as associative and hypothesis-generating. Further large-scale prospective studies are required to better characterize the temporal behavior and clinical relevance of cyclophilin A in preeclampsia.

## Data Availability

The datasets generated and analyzed during this study are not publicly available due to ethical and privacy restrictions but are available from the corresponding author on reasonable request.
